# Optical coherence tomography angiography based prognostic factors and visual outcomes in primary rhegmatogenous retinal detachment after pars plana vitrectomy

**DOI:** 10.1186/s40942-024-00574-0

**Published:** 2024-08-22

**Authors:** Sónia Torres-Costa, Margarida Ribeiro, João Tavares-Correia, Gonçalo Godinho, Pedro Alves-Faria, Manuel Falcão, Amândio Rocha Sousa

**Affiliations:** 1 Ophthalmology Department, Centro Hospitalar Universitário de São João, Porto, Portugal; 2 Department of Biomedicine, Faculty of Medicine, University of Porto, Porto, Portugal; 3 Faculty of Medicine, University of Porto, Porto, Portugal; 4 Ophthalmology Department, Centro Hospitalar de Leiria, Leiria, Portugal; 5 Department of Surgery and Physiology, Faculty of Medicine, University of Porto, Porto, Portugal

**Keywords:** Optical coherence tomography angiography, Rhegmatogenous retinal detachment, Pars plana vitrectomy, Superficial vascular plexus, Vessel density, Visual prognosis

## Abstract

**Purpose:**

To evaluate the visual outcomes and changes in the retinal microcirculation in patients with primary rhegmatogenous retinal detachment (RRD) following successful pars plana vitrectomy (PPV).

**Methods:**

Nine macula-on RRD and 23 macula-off RRD eyes were retrospectively evaluated. Clinical data was collected at admission and 3 months after PPV. Optical coherence tomography angiography (OCTA) was performed 3 months after PPV. Superficial vascular plexus data obtained with OCTA was compared between affected and fellow eyes and according to macular involvement. Quantitative measurements of the superficial retinal capillary associated with the preoperative and intraoperative factors were analyzed.

**Results:**

Overall RRD inner vessel densities (IVD), full vessel densities (FVD), inner perfusion densities (IPD) and full perfusion densities (FPD) were significantly and positively correlated with best corrected visual acuity (BCVA)(*p* = 0.002, *p* = 0.006, *p* = 0.009, *p* = 0.023, respectively). In the macula-off RRD, IVD and FVD were significantly decreased compared with macula-on RRD (*p* = 0.014 and *p* = 0.034, respectively) and significantly correlated with a longer duration and larger extension of detachment. Higher differences of IVD and FVD between the fellow and affected eyes were significantly correlated with worse BCVA in the macula-off subgroup.

**Conclusion:**

Macula-off RRD presented worse OCTA superficial vascular parameters compared with the macula-on group and fellow eyes, which were correlated with a poorer visual outcome and exacerbated by a longer duration and larger extension of the detachment. Macula-off RRD causes not only retinal structural damage but also a reduction in retinal perfusion despite successful anatomical repair.

## Introduction

Rhegmatogenous retinal detachment (RRD) is caused by a retinal tear or hole which allows vitreous fluid outflow into the subretinal space and leads to separation of the neurosensory retina from the retinal pigment epithelium (RPE). This condition affects 1 in each 10 000 individuals every year [1]. Surgical management is required, otherwise severe visual impairment may occur, especially when the macular zone is affected (macula-off RRD).

Pars plana vitrectomy (PPV) is a highly effective surgical procedure for repair of RRD, with anatomical success rates between 80% and 100%. However, the functional outcome regarding postoperative visual acuity remains unpredictable, even in the presence of an apparent normal morphology of the retina in fundus biomicroscopy and normal macular structure in optical coherence tomography (OCT) [2–8]. 

Previous studies identified several prognostic factors for visual outcomes after RRD including macular involvement status, detachment area [9, 10], duration of macular detachment [10, 11], presence of retinal folds and subretinal fluid, [11] and proliferative vitreoretinopathy. Other non-anatomical prognostic factors have been identified, including genetic susceptibility [12, 13], among others. Information regarding the application of OCT angiography (OCTA) parameters as possible visual outcome predictors following a primary RRD episode is recently evolving.

As opposed to fluorescein angiography, OCTA is a novel noninvasive technique for examination and evaluation of retinal and choroidal microcirculation. It provides depth-resolved vascular information without intravenous dye [5, 14, 15]. The retinal vascular planes acquired with OCTA constitute the superficial (SCP), middle (MCP) and deep capillary plexuses (DCP). OCTA technology can analyze vessel density (VD), perfusion density (PD) and foveal avascular zone (FAZ) related parameters, including its area. FAZ area (FAZ-A) has previously been described as a marker of retinal ischemia, as its enlargement occurs due to retinal capillary occlusion [16, 17]. Additionally, enlarged FAZ area has also been described in viral diseases like COVID-19 as an indicator of central retinal hypoxia [18, 19]. Therefore, OCTA provides an innovative, safe, reproducible, and detailed approach to understand the pathogenesis and evolution of retinal diseases. The microvascular changes could explain vision impairment in patients with retinal diseases with otherwise apparent normal fundoscopy and OCT. In this way, OCTA analysis might have an important role in the understanding of RRD pathophysiology. Emerging evidence suggests that RRD may change OCTA values in the macular capillary plexus, affecting postoperative visual outcomes [5] Therefore, OCTA characteristics may comprise possible predictors of functional outcomes in clinical practice after RRD surgery.

The purpose of this study was to analyze macular microvascular changes in the SCP, following a primary RRD managed with PPV irrespective of macular involvement, and to correlate these parameters with postoperative visual outcome.

## Materials and methods

### Participants

This was a single-center study conducted in the Department of Ophthalmology of Centro Hospitalar Universitário de São João. Consecutive patients with primary RRD submitted to successful PPV were included during a period of one year, from January until December of 2019. Exclusion criteria included previous history of RRD, amblyopia, diabetic retinopathy, secondary RRD, scleral buckling, pathologic myopia (< -6 diopters) and poor quality of the OCTA scans. The study was approved by the local ethics committee of Centro Hospitalar Universitário de São João/ Faculdade de Medicina da Universidade do Porto and conducted in accordance with the Helsinki Declaration. A written informed consent was obtained from all individual participants.

### Study design

This was a retrospective comparative case-control study that evaluated macular microvascular changes in the SCP of patients with primary RRD who underwent to PPV. Information was collected at baseline and three months after PPV.

Baseline data included: demographic parameters such as age and sex of the patients; visual symptoms (visual impairment, visual field loss, floaters and photopsia); time since the first visual symptoms were experienced until surgery, best corrected visual acuity (BCVA), intraocular pressure (IOP) and history of myopia and previous cataract surgery. From surgical reports, information regarding the type of surgery performed (PPV vs. PPV combined with cataract surgery), number of tears, location and extension of the retinal detachment, presence of vitreous hemorrhage, presence of proliferative vitreoretinopathy (PVR) and its respective grading (A, B or C, according to the updated Retina Society Classification of 1991) [20], macular status (macula-on vs. macula-off RRDs) and type of tamponade (gas vs. silicone) were collected.

Data collected at the three-month visit included: BCVA, IOP and OCTA parameters.

### OCTA protocol

**Fig. 1:**
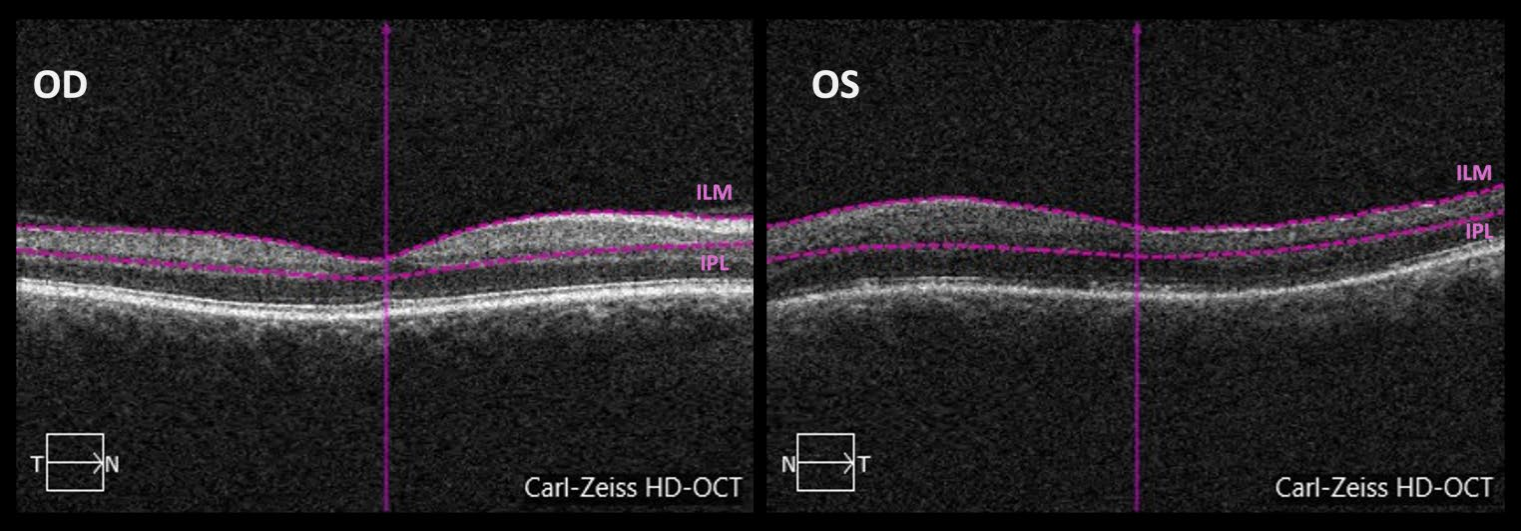
OCTA superficial plexus analysis, it was used a customized segmentation between the inner margin of the internal limiting membrane (ILM) and the outer margin of the inner plexiform layer (IPL). These images are from the fellow eye (right eye, OD) and a macula-off left eye (OS), 3 months after surgery

**Fig. 2:**
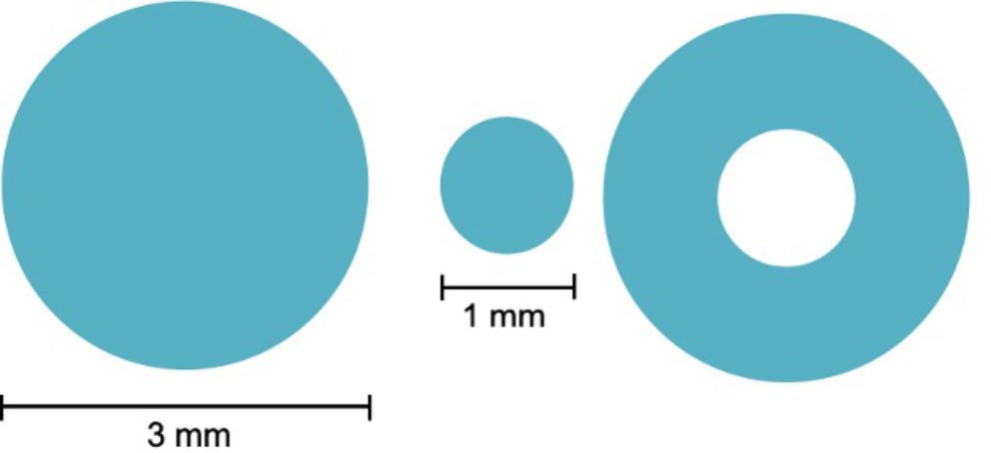
Schematic diagram of OCTA regions of analysis. From left to right are represented the full, central and inner regions. All the aforementioned regions are centered on the fovea

OCTA images were acquired by a specialized technician using CIRRUS™ HD-OCT Model 5000 (Carl Zeiss Meditec, Inc) [14, 21]. The available software (AngioPlex software, version 10.0; Carl Zeiss Meditec, Inc.) was only able to analyze the SCP. *En-face* OCTA images were captured using the customized segmentation between the inner margin of the internal limiting membrane (ILM) and the outer margin of the inner plexiform layer (IPL) (Fig. 1). OCTA examinations were obtained with FastTrac mode, using a standard protocol for macular examination. The 3 × 3 mm scanning pattern, centered on the fovea, was used. OCTA parameters included foveal SCP VD and PD and superficial FAZ-A of the affected and fellow eyes. VD comprises the total length of perfused vasculature per unit area, in a region of measurement (mm/mm^2^). PD is defined as the total area of perfused vasculature per unit area in a region of measurement (%) [22]. VD and PD were measured in three different regions: the full, central and inner regions, as shown in Fig. 2. The central region comprised a circular zone of 1 mm in diameter centered on the fovea. The inner region comprised a ring-shaped area, just peripheral to the central region, with a diameter between 1 and 3 mm. Therefore, VD was represented as central vessel density (CVD, regarding the central region), inner vessel density (IVD, comprising the ring-shaped inner region) and full vessel density (FVD, joining both the central and inner regions). PD was represented as central perfusion density (CPD), inner perfusion density (IPD) and full perfusion density (FPD) respecting the same zoning criteria applied for VD. All OCT-A scans were reviewed to ensure their quality was sufficient ($\:\ge\:7$/10). VD, PD and superficial FAZ-A values were automatically calculated by the OCTA system software. Differences between the fellow eye and the affected eye were represented as “dif” followed by the designation of the OCTA parameter in comparison (i.e. difCVD as the difference of CVD between the fellow eye and the affected eye).

### Statistics

Statistical analysis was performed using the SPSS^®^ Statistical Software (version 26.0 for Windows; SPSS Inc., Chicago, IL., USA). For the qualitative data, frequencies and relative frequencies were calculated. Kolmogorov–Smirnov test and normal probability plots were used to confirm the normal distribution of the data. Variables following the normal distribution were presented as mean and standard deviation, while non-normal distributed variables as median and interquartile range. Parametric or non-parametric tests were used for continuous variables comparison according to the normality of data. Chi-square or Fisher’s exact tests were performed for categorical variables comparison. Spearman’s (rs) correlation test was used to explore the correlation between continuous variables with non-normal distribution. Statistical significance was assumed when *p* < 0.05.

## Results

### Demographics and clinical characterization of RRD

Thirty-two patients were included and 19 of them were males (59.9%). Mean age was 61.5 ± 10.2 years. The most frequently reported symptom was visual impairment (92.9% of the patients). Median BCVA at diagnosis was 20/400 Snellen (range 20/800 to 20/25). Median time from visual impairment until surgery was 6 days (range 3 to 181 days). Among the eyes with RDD, 31.3% of the patients had previously undergone phacoemulsification and 72.7% had non-pathologic myopia (Table 1).

**Table 1:** Baseline characteristics of rhegmatogenous retinal detachment eyes

Variable	*N* = 32
Age at presentation (years; mean ± SD)	61.53 ± 10.15
Male (n, %)	19 (59.40)
**Presenting symptoms (n, %)**	
Reduced visual acuity	26 (92.90)
Floaters	7 (70)
Photopsia	5 (50)
Visual field loss	15 (88.20)
BCVA at diagnosis (feet; median, min - max)	20/400 (20/800 − 20/25)
IOP at diagnosis (mmHg; mean ± SD)	12.75 ± 3.67
Duration of visual impairment until surgery (days; median, min - max)	6 (3–181)
**Risk factors (n; %)**	
Previous cataract surgery	10 (31.30)
Myopia	16 (72.70)
**Macular status (n; %)**	
On	9 (28.10)
Off	23 (71.90)
**RRD Extension (n; %**)	
<90º	8 (25.80)
90º − 180º	12 (38.70)
181º − 270º	5 (16.20)
271º − 360º	6 (19.30)
**RRD Location (n; %)**	
Full	3 (9.70)
Superior	8 (25.80)
Inferior	5 (16.10)
Nasal	2 (6.40)
Temporal	12 (38.70)
Superior + Inferior	1 (3.20)
**Tear number (n; %)**	
1	18 (69.20)
2	4 (15.40)
3	3 (11.50)
5	1 (3.80)
Associated vitreous hemorrhage (n; %)	2 (6.40)
PVR, Grade A/B/C (n; %)	19 (59.40) / 6 (18.75) / 7 (21.8)
**Type of surgery (n; %**)	
PPV	12 (37.50)
PPV + Cataract Surgery	20 (62.50)
**Tamponade (n; %)**	
SF_6_ [22%]	2 (6.50)
C_2_F_6_ [16%]	5 (16.10)
C_3_F_8_ [14%]	23 (74.20)
5,000 cSt Silicone	1 (3.20)

Normal variables are represented by mean ± SD, otherwise stated. Percentage values are expressed as valid percentages (excluding missing values)

SD - standard deviation. min - minimum value. max - maximum value. BCVA - best corrected visual acuity. RRD - Rhegmatogenous retinal detachment. PVR - Proliferative vitreoretinopathy. PPV - Pars plana vitrectomy. SF_6_ - Sulfur hexafluoride. C_2_F_6_ - Hexafluoroethane. C_3_F_8_ - fluoropropane. cSt - Centistokes

Twenty-three (71.90%) patients presented a RRD with macular involvement (macula-off). A retinal detachment larger than 180º was found in 35.5% eyes. The temporal retina was the most commonly affected region (38.70%). Concerning PVR, 40.6% of the patients had grade B or grade C. PPV associated with cataract surgery was performed in 62.50% of patients (Table 2).

**Table 2:** BCVA, IOP and OCTA analysis at three months after surgery according to macular involvement (macula-on Vs. macula-off)

Variable	Macula-on	Macula-off	*p*-value
BCVA 3 months after surgery (feet)	20/32 (20/50 − 20/20)	20/50 (20/200 − 20/20)	0.003*
IOP 3 months after surgery (mmHg)	15.22 (± 2.17)	14.05 (± 3.22)	0.224
CVD (mm/mm²)	11.33 (± 3.73)	10.08 (± 4.92)	0.390
IVD (mm/mm²)	20.19 (± 1.33)	17.47 (± 3.63)	0.014*
FVD (mm/mm²)	19.28 (± 1.29)	16.92 (± 3.57)	0.034*
CPD (%)	23.53 (± 8.90)	20.90 (± 9.07)	0.690
IPD (%)	41.64 (± 5.07)	37.58 (± 9.88)	0.249
FPD (%)	39.92 (± 5.25)	36.34 (± 9.60)	0.285
FAZ-A (mm²)	0.28 (± 0.16)	0.32 (± 0.25)	0.922

Normal variables are represented by mean ± standard deviation and non-normal variables are represented by median [minimum value – maximum value] and significance (p). Statistically significant values (*p* < 0.05) are in bold followed by (*)

BCVA - Best corrected visual acuity. IOP - Intraocular pressure. mmHg - millimeters of mercury. OCTA - Optical coherence tomography angiography. CVD - Central vessel density. IVD - Inner vessel density. FVD - Full vessel density. CPD - Central perfusion density. IPD - Inner perfusion density. FPD - Full perfusion density. FAZ-A - Foveal avascular zone area

### Functional outcomes after surgery

At 3 months, median BCVA was significantly increased in the macula-on subgroup compared with the macula-off subgroup (20/32 vs. 20/50, respectively; *p* = 0.003).

### OCTA analysis at 3 months after surgery

In the overall analysis (macula-on and macula-off RRD), the IPD in the affected eye was inferior to the fellow eye (38.72 ± 8.91% vs. 39.56 ± 7.42%, respectively; *p* = 0.030). No other differences were found in the overall analysis concerning the OCTA superficial plexus parameters.

When performing a subgroup analysis by macular status, in the macula-off subgroup, IPD was significantly decreased in the affected eye compared with the fellow eye (37.58 ± 9.88% vs. 38.35 ± 8.15%, respectively; *p* = 0.034). In the macula-on subgroup, no significant differences were found between the affected eye and fellow eye.

**Fig. 3:**
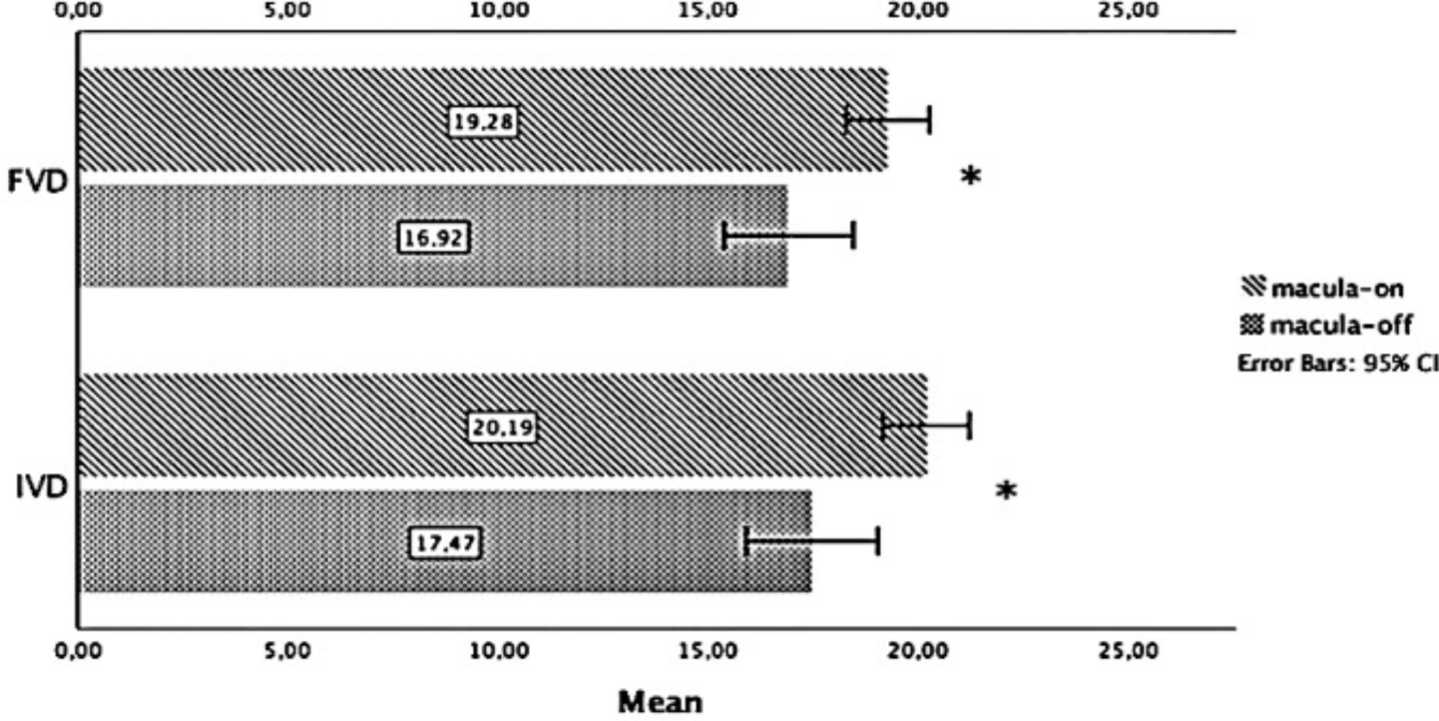
Comparison of IVD and FVD between macular subgroups 3 months after PPV. Values are expressed as mean in units of mm/mm². The error bars represent a 95% confidence interval (CI). Statistically significant values (*p* < 0.05) are represented with (*). IVD - Inner vessel density. FVD - Full vessel density. PPV - Pars plana vitrectomy

IVD (20.19 ± 1.33 mm/mm^2^ vs. 17.47 ± 3.63 mm/mm^2^, respectively; *p* = 0.014) and FVD (19.28 ± 1.29 mm/mm^2^ vs. 16.92 ± 3.57 mm/mm^2^, respectively; *p* = 0.034) were increased in the macula-on eyes compared with the macula-off eyes (Table 3; Fig. 3).

**Table 3:** Macular subgroup correlation between OCTA and clinical parameters

		CVD	IVD	FVD	CPD	IPD	FPD	FAZ-A
**Macula-on**								
BCVA 3 months after surgery	Spearman	0.589	0.148	0.221	0.356	0.135	0.135	-0.319
	p	0.124	0.726	0.599	0.387	0.750	0.750	0.441
RRD extension	Spearman	0.675	0.155	0.282	0.540	0.235	0.209	-0.626
	p	0.066	0.713	0.498	0.167	0.576	0.620	0.097
Duration visual impairment-surgery	Spearman	-0.201	-0.022	0.079	0.122	0.360	0.358	0.087
	p	0.604	0.955	0.841	0.754	0.341	0.343	0.823
PVR	Spearman	0.338	-0.124	-0.064	0.119	-0.312	-0.347	-0.557
	p	0.374	0.750	0.870	0.761	0.414	0.360	0.119
**Macula-off**								
BCVA 3 months after surgery	Spearman	0.224	0.474*	0.433	0.232	0.422	0.401	-0.195
	p	0.330	0.030*	0.050	0.312	0.056	0.072	0.424
RRD extension	Spearman	-0.346	-0.514*	-0.474*	-0.318	-0.184	-0.159	0.000
	p	0.106	0.012*	0.022*	0.139	0.401	0.469	1.000
Duration visual impairment-surgery	Spearman	-0.294	-0.704*	-0.579*	-0.385	-0.559*	-0.474*	-0.073
	p	0.208	0.001*	0.007*	0.094	0.010*	0.035*	0.782
PVR	Spearman	-0.107	-0.403	-0.312	-0.087	-0.230	-0.144	-0.205
	p	0.628	0.057	0.147	0.694	0.290	0.513	0.399

Univariate analysis using Spearman correlation. Values are expressed as Spearman correlation coefficients and significance (p). Statistically significant values (*p* < 0.05) are in bold followed by (*)

OCTA - Optical coherence tomography angiography. BCVA - Best corrected visual acuity. CVD - Central vessel density. IVD - Inner vessel density. FVD - Full vessel density. CPD - Central perfusion density. IPD - Inner perfusion density. FPD - Full perfusion density. FAZ-A - Foveal avascular zone area. RRD - Rhegmatogenous retinal detachment. PVR - Proliferative vitreoretinopathy

### Correlations between clinical and microvascular parameters

Three months after surgery, in the overall analysis, we observed a statistically significant moderate positive correlation between age and FAZ-A (rs = 0.421, *p* = 0.026), and between BCVA 3 months after surgery and IVD (rs = 0.541, *p* = 0.002), FVD (rs = 0.502, *p* = 0.006), IPD (rs = 0.475, *p* = 0.009) and FPD (rs = 0.421, *p* = 0.023). A moderate negative correlation was verified between the duration of visual impairment and IVD (rs=-0.411, *p* = 0.027) and between RRD extension and both IVD (rs=-0.527, *p* = 0.002) and FVD (rs=-0.530, *p* = 0.002). PVR and IVD were negatively (although weakly) correlated (rs=-0.397, *p* = 0.024). No significant correlation was found in the overall analysis between age, gender, duration of visual impairment before surgery, IOP at diagnosis, number of tears, RRD extension and location, PVR, vitreous hemorrhage, type of tamponade, presence of myopia, IOP at 3 months after surgery and the remaining microvascular SCP parameters. (Table 4).

**Table 4:** Correlation between OCTA parameters differences and BCVA at 3 months

		BCVA 3 months after surgery	
		Macula-on	Macula-off
difCVD	Spearman	-0.596	-0.127
	p	0.090	0.519
difIVD	Spearman	-0.150	-0.439*
	p	0.700	0.019*
difFVD	Spearman	-0.519	-0.448*
	p	0.152	0.017*
difCPD	Spearman	-0.392	-0.131
	p	0.297	0.506
difIPD	Spearman	0.102	-0.367
	p	0.794	0.055
difFPD	Spearman	0.260	-0.326
	p	0.500	0.090
difFAZ-A	Spearman	0.462	0.290
	p	0.211	0.160

Univariate analysis using Spearman correlation. Values are represented as Spearman correlation coefficients and significance (p). Statistically significant values (*p* < 0.05) are in bold followed by (*)

OCTA - Optical coherence tomography angiography. BCVA - best corrected visual acuity. difCVD - Difference in mean value of central vessel density between fellow and affected eyes. difIVD - Difference in mean value of inner vessel density between fellow and affected eyes. difFVD - Difference in mean value of full vessel density between fellow and affected eyes. difCPD - Difference in mean value of central perfusion density between fellow and affected eyes. difIPD - Difference in mean value of inner perfusion density between fellow and affected eyes. difFPD - Difference in mean value of full perfusion density between fellow and affected eyes. difFAZ-A - Difference in mean value of foveal avascular zone area between fellow and affected eyes

In the subgroup analysis, the extension of detachment was negatively and significantly correlated with both IVD (rs=-0.514, *p* = 0.012) and FVD (rs=-0.474, *p* = 0.022) in the macula-off subgroup. In the macula-off subgroup, the duration of visual impairment until surgery was negatively correlated with IVD (rs=-0.704, *p* = 0.001), FVD (rs=-0.579, *p* = 0.007), IPD (rs=-0.559, *p* = 0.010) and FPD (rs=-0.474; *p* = 0.035). BCVA was positively correlated with IVD (rs = 0.474; *p* = 0.030). No significant correlations were observed in the macula-on subgroup.

When analyzing the differences of superficial microvascular OCTA parameters between the affected and non-affected eyes, a negative correlation between difIVD and BCVA (rs=-0.439, *p* = 0.019) and between difFVD and BCVA (rs=-0.448, *p* = 0.017) were observed in the macula-off subgroup. No statistically significant differences were observed in the macula-on subgroup.

## Discussion

In our study, we evaluated the microvascular OCTA parameters of superficial vascular plexus (IVD, FVD, IPD and FPD) and functional outcomes (BCVA) after primary RRD, according to macular involvement, following uncomplicated PPV with postoperative anatomical success.

In the overall analysis, the differences found in the superficial microvascular parameters and the association between BCVA and the OCTA parameters could be explained by the macula-off subgroup, since the majority of patients (23 patients, 71.90%) presented macular detachment and no correlations were found in the macula-on group in the subgroup analysis.

No differences were found between the affected and the fellow eye in the macula-on group, regarding any of the macular vascular parameters, since macular vascular disturbance and ischemia does not occur in eyes classified as macula-on in fundus biomicroscopy and in OCT. However, Barca and colleagues [23] verified that even in eyes without macular involvement, early decrease of the macular superficial vascular density may be detectable, with a recovery at 6 months of follow-up. In our study, if any microvascular alteration had occurred in the macula-on RRD eyes, this was resolved at 3 months postoperatively, but not in the macula-off group, with worse superficial microvascular parameters and poorer final visual acuity.

In fact, previous studies demonstrated that macular status was associated with visual prognosis [5, 9, 10, 23]. In the macula-off subgroup, the postoperative OCTA quantitative parameters IVD and FVD were significantly decreased when compared with the macula-on subgroup. Higher values of difIVD and difFVD (which means lower IVD and FVD in macula-off eyes comparing with the fellow eyes) and lower values of IVD were correlated with poorer BCVA 3 months after uncomplicated PPV with anatomical reattachment.

Furthermore, the duration of macular detachment is another main preoperative predictor of postoperative VA [10, 11]. We observed that longer duration of macular detachment was significantly correlated with worse BCVA at 3 months. The duration of detachment was itself inversely correlated with several OCTA biomarkers, like IVD, FDV, IPD and FPD. Christou et al. also observed that macular capillary plexus microcirculation was more vulnerable to changes and tissue destruction caused by RRD with macular involvement in chronic than in recent onset cases [5]. Moreover, the extension of RRD was also significantly correlated with macular microvascular status (IVD and FVD). A longer RRD duration and a larger detachment extension could induce greater ischemic damage and significant microvascular changes. The subretinal fluid (SRF) limits free diffusion of oxygen from the choriocapillaris to the photoreceptor layer, leading to tissue hypoxia and nutrient deprivation. The levels of inflammatory and vascular mediators including prostaglandins, cytokines and endothelin-1 in the SRF are also increased. Muller cell activation and promotion of vasoconstriction are other of the proposed mechanisms for the vascular changes [5, 23–25]. Furthermore, macular vascular flow reduction may be a consequence of an autoregulatory mechanism of reversible vasoconstriction due to hypoxia, which could become sufficiently severe and prolonged following long-duration and extensive retinal detachment, leading to structural vascular changes and capillary dropout [5, 23]. 

Regarding other OCTA quantitative parameters, in our study, CVD and CPD were not significantly affected by macular status or correlated with BCVA. Thus, it could indicate the existence of a central vascular region more resistant to the disruptive detachment forces. In fact, a previously described anatomic structure called foveal ring, which consists of the connection point between the three retinal capillary plexuses (and corresponds to the edge of the FAZ) could offer more resistance to a process of hemodynamic instability triggered by the retinal detachment [26]. Conversely, the foveola, the thinnest region and responsible for the highest visual acuity, characterized in this study by CVD and CPD, is independent of the vascularization provided by the SCP and, in contrast, it is fully dependent on the choriocapillary plexus (CCP) perfusion. However, it would be plausible to conceive that there could be some permanent changes in foveal microvascular parameters as sequels of retinal detachment. Nonetheless, when comparing the macula-off eyes with their fellow eyes, only IPD was significantly decreased in the affected eye. Nevertheless, as previously demonstrated by Wang et al., retinal microvasculature tends to recover in some extent over a 12-week follow-up period, after successful surgical repair [27]. 

This study has limitations. Firstly, we must point its retrospective nature. Secondly, we investigated the parameters of FAZ, VD and macular perfusion in the SCP and not in the deep nor the choriocapillary plexus due to software limitations that only allowed direct extraction of microvascular parameters in the SCP. Nonetheless, it is hypothesized that in a recent onset disease such as retinal detachment, the SCP is the most affected plexus because it may be the first vascular layer involved due to its greater density in arterioles and its smooth muscle, leading to a stronger and faster contraction with vascular flow, resistance and vessel density changes [23]. Wang et al. and Tsen et al. found similar results, not only comprising the SCP, but also in the deep and choriocapillaris layers [27, 28]. Hong et al. also found that the postoperative subfoveal VD of the CCP was positively correlated with BCVA in macula-off RRD [29]. Thirdly, the sample size was relatively small. Fourthly, we could not disregard the existence of previously described confounding factors including spherical equivalent (although we have excluded patients with spherical equivalent < -6D) and axial length, medication and systemic hypertension, which may influence the microvascular evaluation with OCTA [30, 31] and, possibly, the visual outcome. Moreover, the postoperative follow-up period was relatively short. Our patients were imaged shortly after presentation with a follow-up period of 3 months after surgery, similar to other studies [5, 23, 27]. It would be of great benefit to continue the examination of the patients for a longer period to confirm our results. Lastly, the differences observed in the IPD between the affected and fellow eyes in the overall analysis and among the macula-off group, although statistically significant, were small. This finding should be interpreted concerning its relevance in clinical practice.

In conclusion, we found the decrease of IVD and FVD to be correlated with a worse BCVA exclusively in macula-off eyes, three months after uncomplicated repair surgery with anatomical success. Macular detachment would be the trigger for these vascular abnormalities, which are exacerbated by a longer duration and larger extension of the detachment. These correlations found between BCVA and superficial microvascular changes allow a deeper comprehension of the physiopathology behind vision impairment in macula-off eyes after RRD. Further prospective randomized studies with a larger number of patients and a longer follow-up period should be performed to support our findings and to assess long term visual outcomes.

## Data Availability

All data generated or analysed during this study are included in this published article.
